# Sexually Dimorphic Regulation of EET Synthesis and Metabolism: Roles of Estrogen

**DOI:** 10.3389/fphar.2018.01222

**Published:** 2018-10-29

**Authors:** An Huang, Dong Sun

**Affiliations:** Department of Physiology, New York Medical College, Valhalla, NY, United States

**Keywords:** epoxyeicosatrienoic acids, soluble epoxide hydrolase, sex, estrogen, pulmonary hypertension

## Abstract

Epoxyeicosatrienoic acids (EETs) are metabolites of arachidonic acid via cytochrome P450 (CYP)/epoxygenase and are hydrolyzed by soluble epoxide hydrolase (sEH). Circulating and tissue levels of EETs are controlled by CYP (EET synthesis) and sEH (EET degradation). Therefore, both increases in CYP activity and decreases in sEH expression potentiate EET bioavailability, responses that prevail in the female sex as a function of estrogen. This mini review, based on subtitles listed, briefly summarizes studies focusing specifically on (1) female-specific potentiation of CYP/epoxygenase activity to compensate for the endothelial dysfunction; and (2) estrogen-dependent downregulation of sEH expression, which yields divergent actions in both systemic and pulmonary circulation, respectively.

**Estrogen-Potentiating EET Synthesis in Response to Endothelial Dysfunction:** This section summarizes the current understanding regarding the roles of estrogen in facilitating EET synthesis in response to endothelial dysfunction. In this regard, estrogen recruitment of EET-driven signaling serves as a back-up mechanism, which compensates for NO deficiency to preserve endothelium-dependent vasodilator responses and maintain normal blood pressure.

**Estrogen-Dependent Downregulation of *Ephx2*/sEH Expression:** This section focuses on molecular mechanisms responsible for the female-specific downregulation of sEH expression.

**Roles of EETs in Systemic Circulation, as a Function of Estrogen-Dependent Downregulation of sEH:** This section summarizes studies conducted on animals that are either deficient in the *Ephx2* gene (sEH-KO) or have been treated with sEH inhibitors (sEHIs), and exhibit EET-mediated cardiovascular protections in the cerebral, coronary, skeletal, and splanchnic circulations. In particular, the estrogen-inherent silencing of the *Ephx2* gene duplicates the action of sEH deficiency, yielding comparable adaptations in attenuated myogenic vasoconstriction, enhanced shear stress-induced vasodilation, and improved cardiac contractility among female WT mice, male sEH-KO and sEHI-treated mice.

**Roles of Estrogen-Driven EET Production in Pulmonary Circulation:** This section reviews epidemiological and clinical studies that provide the correlation between the polymorphism, or mutation of gene(s) involving estrogen metabolism and female predisposition to pulmonary hypertension, and specifically addresses an intrinsic causation between the estrogen-dependent downregulation of *Ephx2* gene/sEH expression and female-susceptibility of being pulmonary hypertensive, a topic that has never been explored before. Additionally, the issue of the “estrogen paradox” in the incidence and prognosis of pulmonary hypertension is discussed.

## Introduction

It is established that oxidative metabolism of arachidonic acid through the cyclooxygenase (COX) and lipoxygenase pathways to biologically activate eicosanoids plays a critical role in the regulation of pathophysiological processes. To date, a dubbed “third pathway” of the cytochrome P450 (CYP)/epoxygenase system has come to the forefront of research with aims to evaluate the pathophysiological significance of its biologically active mediators, epoxyeicosatrienoic acids (EETs) (Roman, 2002). EETs possess cardiovascular protective properties in the systemic circulation via endothelium-derived hyperpolarizing factor (EDHF)-based vasodilator responses (Archer et al., 2003; Fleming, 2004; Huang et al., 2004, 2005) to lower blood pressure in both physiological (Imig, 2012; Sun et al., 2014) and pathological conditions (Lee et al., 2010; Ma et al., 2013). The contribution of EETs toward the cardiovascular protection can be controlled by soluble epoxide hydrolase (sEH), an enzyme that hydrolyzes EETs to their biologically inactive diols (dihydroxyeicosatrienoic acids, DHETs) (Newman et al., 2005). As such, either the potentiation of EET synthesis or the reduction of EET metabolism is able to increase EET bioavailability, and therefore, both CYP/epoxygenase and sEH can be therapeutic targets for cardiovascular diseases. More importantly, both enzymatic activities are regulated by female hormones/estrogens, leading to a sex disparity in the presentation of EET-mediated contributions. In general, beneficial actions of EETs in the cardiovascular system have been well reviewed (Roman, 2002; Imig, 2012), however, the sexually dimorphic phenotype, in terms of female-favorable contributions of EETs is much less addressed. Thus, this mini-review will summarize studies from the authors’, as well as others’ laboratories, focusing on (1) female-specific potentiation of CYP activity to compensate for the endothelial dysfunction; and (2) estrogen-dependent suppression of sEH expression that yields divergent actions in the systemic and pulmonary circulation, respectively.

## Estrogen-Favorable EET Synthesis in Response to Nitric Oxide Deficiency

Cytochrome P450 are encoded by a complex superfamily of genes; they are located in the endoplasmic reticulum and add an epoxide across one of the four double bonds of arachidonic acid to produce four EET regiosomers: 5,6-EET, 8,9-EET, 11,12-EET and 14,15-EET. The CYP2C and CYP2J families are responsible for the majority of EET generation in mammals (Imig, 2012). Specifically, CYP2C29 and CYP2C7 are EET synthasase in mouse and rat vascular endothelium, and express predominantly in female vessels deficient in NO synthesis (Sun et al., 2010, 2011).

One of rationales for investigating CYP/epoxygenase function is its compensatory nature, characterized by the fact that the enzymatic activity and its contribution to the regulation of cardiovascular function are dampened under physiological conditions, and become discernible in most instances, only with endothelial dysfunction, manifested as impaired NO bioavailability. Therefore, most *in vitro* studies aiming to evaluate CYP activities were performed in the presence of inhibitors of endothelial nitric oxide synthase (eNOS) and COX. More intriguingly, the CYP/EET-evoked compensatory action exerts in a female favorable manner, as indicated by the evidence that in eNOS and COX-1 double knockout (KO) mice, EET-mediated responses via an EDHF-based event contribute significantly to the preservation of endothelium-dependent relaxation, coinciding with normal blood pressure in female animals (Scotland et al., 2005), with little of this compensation in their male counterparts that display hypertension, associated with impaired endothelium-dependent vasodilations (Brandes et al., 2000). The same responsive pattern was also observed in the high fructose-induced metabolic syndrome or chronic insulin-loading animal models, where only hyperinsulinemic male rats, not females, developed hypertension, even though both sexes displayed endothelial dysfunction (Galipeau et al., 2002; Vasudevan et al., 2005); moreover, female ovariectomy (OV) prevented, and OV with estrogen replacement (OVE) restored the normotension (Galipeau et al., 2002; Song et al., 2005). These findings clarify estrogen as an essential player in the compensation against endothelial dysfunction (deficiency of NO and/or PGs), via perhaps, recruiting EET/EDHF-dependent signaling.

In the microcirculation, estrogen, in response to NO deficiency, affords protection via unveiling the EET/EDHF-mediated pathway as a back-up mechanism, to maintain normal microcirculatory resistance. For instance, in female eNOS-KO mice and female rats treated with L-NAME, estrogen via activation of estrogen receptors (ERs), evokes a solely EET-mediated response that fully preserves shear stress-induced vasodilation (SSID, one of the most important local regulators in the control of microcirculatory resistance) (Huang et al., 2001a,b; Wu et al., 2001), reminiscent of a significantly smaller magnitude of SSID mediated by COX-derived prostaglandins (PGs) in male eNOS-KO and L-NAME treated counterparts (Sun et al., 1999, 2006). Therefore, the female phenotype of SSID is defined as augmented vasodilator responses mediated by EETs in an EDHF-based approach, as a function of either decreased NO, or increased EET bioactivities (Huang and Kaley, 2004), highlighting further, a reverse interaction between the two endothelial mediators (NO vs. EETs). The female phenotypic SSID (EET-mediation) can be changed to male phenotype of SSID (PG mediation) when gonad-intact females are ovariectomized (Huang et al., 2001b); vice versa, *in vitro* exposure of male vessels to a physiological concentration of estrogen enables to elicit a female phenotype of SSID (Huang et al., 2004). Thus, in the deficiency/impairment of NO bioactivity, vascular release of EETs to maintain a normal endothelial sensitivity to shear stress is dependent of estrogen and occurs via an ER-mediated activation of a PI3K/Akt pathway to upregulate CYP2C29 and CYP2C7 genes (Huang et al., 2004; Sun et al., 2011).

## Estrogen-Dependent Downregulation of *Ephx2*/sEH Expression

Mammalian sEH is encoded by the *Ephx2* gene and extensively expressed in multiple organs/tissues including vasculatures; it converts epoxides to diols by adding water to open the epoxide, thus inactivating EETs (Harris and Hammock, 2013). The majority of cardiovascular protective actions elicited by pharmacological inhibition of sEH activity using sEH inhibitors (sEHIs) or genetic deletion of the *Ephx2* gene have been ascribed to be due to increases in circulating and tissue/cellular EET levels (Fang et al., 2004; Deng et al., 2011).

Noteworthily, the estrogen-potentiation of EET production takes place primarily in the presence of endothelial dysfunction, whereas estrogen-downregulation of sEH occurs inherently in physiological conditions. The identification of sexual dimorphism of sEH was originally reported around the 1980’s, where sEH activity was found to be remarkably higher in organs/tissues of male and OV female mice in comparison to intact females (Denlinger and Vesell, 1989; Pinot et al., 1995), and further validated by a female-specific downregulation of sEH expression (Zhang et al., 2009; Kandhi et al., 2015; Froogh et al., 2016; Qin et al., 2016). This is reminiscent of the phenomenon known as the “male-specific hypotensive response to sEH deficiency,” where the deletion of the *Ephx2* gene in male mice elicited a significant reduction in blood pressure, with minimal hypotensive effects on female mice (Sinal et al., 2000). We found that KO of the *Ephx2* gene (sEH-KO) or treatment with sEHIs in male mice reduced their blood pressure to the level comparable to that of wild type (WT) females; in the latter, disruption of the *Ephx2* gene further reduced blood pressure but with significantly smaller decrement than in male counterparts (Kandhi et al., 2015; Qin et al., 2015a; 2016; Froogh et al., 2016). This dose-dependent-like phenomenon implies that females may heritably possess a mechanism that imitates an action caused by the deletion of the *Ephx2* gene in males, making females less sensitive to an additional disruption of the gene. By using *in vivo* and *in vitro* models, we demonstrate that estrogen, through ERs, methylates the *Ephx2* gene promoter to silence its transcriptional activity, a response that involves multiple transcription factor-driven regulatory signaling (Yang et al., 2018). This study provides mechanistically based explanations for the sexually dimorphic expression of sEH and all the consequences arising therefrom, that will be discussed in the forthcoming sections.

## Roles of EETs in Systemic Circulation, as a Function of Estrogen-Dependent Downregulation of sEH

Female-specific downregulation of sEH expression stabilizes EETs and functionally potentiates EET bioavailability.

In the cerebral circulation, studies using animal models of ischemia demonstrated that estrogen suppression of sEH was responsible for the female-favorable protection against cerebral ischemic damages in an EET-dependent manner (Fairbanks et al., 2012; Davis et al., 2013).

In the coronary circulation, our laboratories have provided evidence indicating sex-different adaptation of cardiac performance, by conducting experiments on Langendorff-perfusion preparations. In physiological conditions, challenged with same increases in preload, female hearts displayed significantly greater cardiac contractility, associated with enhanced coronary blood flow and lower vascular resistance compared to male hearts (Qin et al., 2016). Isolated coronary arteries from female hearts exhibited significantly attenuated pressure-induced myogenic vasoconstriction compared to male arteries, responses that were prevented by 14,15-EEZE (a putative inhibitor of EETs) (Froogh et al., 2016; Qin et al., 2016). These female-specific adaptations were also observed in male sEH-KO mice, implying that estrogen downregulation of sEH duplicates actions of *Ephx2* deletion, yielding identical patterns of attenuated coronary myogenic responses, enhanced coronary perfusion and improved cardiac contractility, along with similar cardiac EET metabolic profiles (a great ratio of EETs/DHETs) among female WT, male sEH-KO mice and male WT mice treated with sEHIs (Sun et al., 2014; Qin et al., 2015b). In pathological conditions, Seubert’s group using cardiac ischemia models provided strong evidence indicating an EET-driven protection against ischemia/reperfusion-induced cardiac injury in sEH deficient animals (Chaudhary et al., 2009).

In the skeletal muscle and splanchnic circulations, isolated arterioles from female WT mice exhibited significantly greater magnitude of EET-mediated SSID, accompanied with attenuated arteriolar tone than those of male WT controls, responses that were also elicited in vessels isolated from male sEH-KO mice (Sun et al., 2014; Qin et al., 2015a).

Collectively, the evidence of estrogen-dependent suppression of *Ephx2*/sEH expression provides a novel mechanistic explanation, in addition to the estrogen potentiation of NO-mediated responses (Huang and Kaley, 2004), for the better cardiac performance and lower incidence of ischemic cardiovascular diseases in women than men.

## Roles of Estrogen-Driven EET Production in Pulmonary Circulation

The systemic circulation is benefited from EETs, which then, creates a question as to whether the increase in pulmonary EETs is a “friend or foe?” In addition to the typical feature of low oxygenated blood in the pulmonary artery (PA), there are other two unique features in the pulmonary circulation that differ from the systemic circulation: (1) hypoxia pulmonary vasoconstriction (HPV) (Sylvester et al., 2012) that is reminiscent of hypoxia-induced vasodilation in systemic vasculatures (Busse et al., 1983) and (2) EET-induced pulmonary vasoconstriction via perhaps, depolarizing PA smooth muscle cells (SMC) (Zhu et al., 2000; Kandhi et al., 2016) in contrast to EET-induced vasodilation in systemic vasculatures via hyperpolarizing vascular SMC. Although currently, there is no specific explanation for the divergent responsiveness to hypoxia and EETs in the pulmonary circulation, both features seem to be relevant to estrogens, which therefore, sheds light upon the categorization of pulmonary hypertension (PH) as a disease with female-specific prevalence (Miller, 2012).

Thus, human studies show a female to male ratio of 4.3:1 among the total PH patients (Walker et al., 2006), and 4.1:1 in the idiopathic PH (IPAH) subcategory (Badesch et al., 2010). This sexual dimorphism in PH is evoked at least in part, by estrogen, as evidenced by a high PH prevalence in women who have taken oral contraceptives (Kleiger et al., 1976), received hormone replacement therapy (Sweeney and Voelkel, 2009) or had enhanced aromatase activity (Roberts et al., 2009). Also, male IPAH patients exist with significantly higher plasma estrogen levels, or a greater ratio of estrogen to testosterone than healthy males (Wu et al., 2018).

Clinical studies provide correlations between the polymorphism of gene(s) involving estrogen metabolisms and the female predisposition to PH (Austin et al., 2009). In general, the mutation in the *BMPR2* gene turns out to be one of the most important genetic-based alterations responsible for the sex-bias in IPAH (Morse et al., 2001; Austin et al., 2013; Dempsie et al., 2011; White et al., 2011), as the penetrance of PH among *BMPR2* mutation carriers shows a 42% penetrance in females vs. 14% in males (Larkin et al., 2012). Female *BMPR2* mutation carriers with PH exhibit a ten-fold reduction in *CYP1B1* gene expression (West et al., 2008; White et al., 2012), followed by altered estrogen metabolism, manifested by a significantly lower ratio of 2-OHE½ to 16α-OHE_1_ (Austin et al., 2009). This estrogen dysmetabolism shifts the balance away from 2-OHE½-induced anti-mitogenic effects toward 16α-OHE_1_-stimulated pulmonary mitogenic and genotoxic pathways (Austin et al., 2013). Direct binding of ERα to the promoter of *BMPR2* gene silences its expression, which disrupts downstream signaling of bone morphogenetic protein-dependent ligand binding, kinase activation and heteromeric dimer formation etc. (Lane et al., 2000; Austin et al., 2012; Johansen et al., 2016).

To date, there is little attention paid to the intrinsic causation between the estrogen-driven physiological downregulation of *Ephx2* gene/sEH and female-susceptibility to be pulmonary hypertensive, a topic that is being investigated in our laboratories. As reported, increases in pulmonary EETs caused by estrogen downregulation of sEH, knockdown of sEH and treatment with sEHIs propel HPV and promote elevation of PA pressure in response to acute hypoxia (Keseru et al., 2010; Kandhi et al., 2015, 2017). Underlying mechanisms responsible for the EET-dependent potentiation of HPV and hypoxia-induced pulmonary hypertension (HPH) remain elusive; however, roles of vasoconstrictor prostanoids and Rho kinase in this process have emerged, as shown that enhanced hypoxic responses were prevented by 14,15-EEZE, and by inhibition of COXs and Rho kinase, respectively (Keseru et al., 2008; Kandhi et al., 2017). Additionally, the membrane translocation of a TRPC6-V5 fusion protein within PASMC was sensitive to 14,15-EEZE, and hypoxia-induced EET-mediated increases in pulmonary pressure failed to be elicited in mouse lungs that were deficient in TRPC6 (Keseru et al., 2008). Thus, the interaction among estrogen/ERs, sEH/EETs and hypoxia/TRPC6/Rho kinase/PGs works reciprocally, forming a feedback loop in a pattern of cause and result for one another, to elevate PA pressure.

**FIGURE 1:**
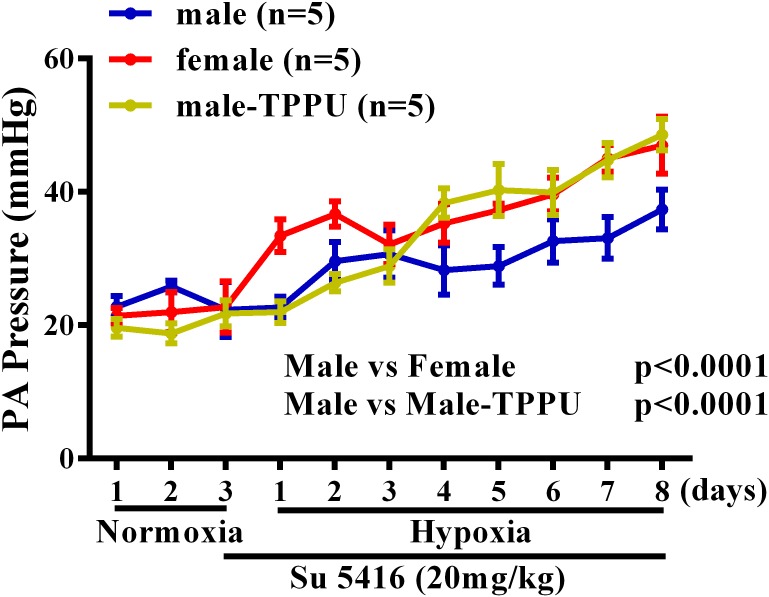
Changes in pulmonary artery (PA) pressure before (normoxia) and after exposure of male (blue) and female (red) mice, and male rats treated with TPPU (green) to hypoxia for 8 days.

Furthermore, the sex disparity during the development of HPH was evaluated by using radio-telemetry to dynamically monitor changes in rat PA pressure. Figure 1 shows that under a comparable basal/normoxic PA pressure, male and female rats displayed a time-dependent elevation of PA pressure in response to hypoxia, which, however, occurred earlier accompanied with greater magnitude in females than males, revealing female oversensitivity to hypoxia. This hypoxic responsiveness in female rats was also observed in sEHI-treated male rats, indicating the role of EETs in the event. Noteworthily, during the process of HPH development, hypoxia *per se*, enables to stimulate EET synthesis (Michaelis et al., 2005) and suppress sEH expression in a sex-independent manner (Petruzzelli et al., 1992), which exacerbate EET-mediated HPV in both genders. Alternatively, when PH is ultimately established, female patients paradoxically exhibited less impairment in right ventricle (RV) function, indicating better PH prognosis compared to males (Mair et al., 2014).

**FIGURE 2:**
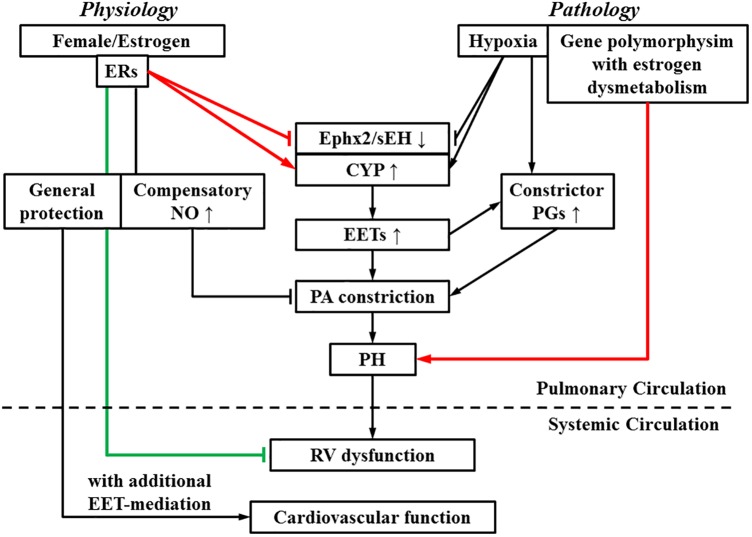
During the process of switch from a physiologically based downregulation of sEH by estrogen/estrogen receptors (ERs) to the pathological development of pulmonary hypertension (PH), multiple pathological stimuli operate in concert, to interrupt the normal physiological balance, tipping the balance toward PA constrictor axis. As indicated, pathological increases in EETs, as a function of upregulation of EET syntheses (CYP) or suppression of *Ephx2*/sEH elicit PA constriction and promote actions of constrictor prostaglandins (PGs), resulting in an EET-potentiation of PH, which eventually impairs right ventricular (RV) function. On the other hand, RV dysfunction is alleviated by estrogen. The systemic circulation is also benefited by estrogen- and EET-mediated protections. Arrow-head indicates promotion. Flat-head indicates suppression. Estrogen-promotion of incidence and -improvement of prognosis of PH are demarcated by lines designated in red and green respectively.

A lack of consensus regarding to roles of estrogen in PH is presumably, due to the presence of the “estrogen paradox” that is characterized by divergent actions (detrimental and beneficial) of estrogens in the incidence and prognosis of PH (Lahm et al., 2014). Figure 2 interprets “estrogen paradox” to mean that *Ephx2*/sEH and CYP are intimately involved in the pathogenesis of HPH, via being targeted and dysregulated by estrogen and hypoxia to increase pulmonary EET bioavailability. Female PAs are capable of maintaining a normal pressure in response to physiological increases in pulmonary EETs due to the presence of compensatory balancing mechanisms such as estrogen-upregulation of eNOS/NO, but bear hyper-responsiveness to acquired pathological challenges such as hypoxia or altered estrogen metabolism, leading to the female susceptibility to PH. Alternatively, all types of PH regardless of their specific etiologies, undergo/share a common progressive process that involves multiple pathological alterations including but not limited to, the endothelial dysfunction, enhanced oxidative stress and inflammation, vascular remodeling and formation of occlusive lesions, leading to RV hypertrophy and dysfunction, and eventually right heart failure (Rabinovitch, 2012), whereas, all of these pathological alterations can be challenged against by estrogens. For instance, in sugen-hypoxia (SuHx-HP)-induced PH, estrogen improves RV function via inotropic effects on myocardium (Liu et al., 2014) and restoration of conduit PA compliance (Liu et al., 2015). In monocrotaline (MCT)-induced PH, estrogen prevents MCT-induced impairment of antioxidant capacity to preserve myocardial function (Bal et al., 2013). This points to a non-specific pattern of estrogen-driven improvement of PH prognosis, which is neither selectively triggered by a specific model of PH, nor does it direct particular target(s), and provides explanations for the better prognosis with higher survival rate in female than male PH patients.

Currently, clinical trials designed to evaluate the protection of sEHIs if any, for patients with chronic obstructive pulmonary diseases (COPD) reveled that GSK alleviated endothelial dysfunction in COPD patients (Lazaar et al., 2016; Yang et al., 2017). Since HPH, as well as COPD, is associated with downregulation of sEH and upregulation of EET production, the improvement of endothelial function elicited by GKS in COPD patents may not be purely, mediated by the inhibition of sEH *per se*, but rather by alternative pathways. Indeed, in addition to targeting sEH, sEHIs are capable of binding with other enzymes due to the presence of multi-target ligands. For instance, PTUPB is a tight COX-2 binder (Hwang et al., 2018) and TPPU selectively inhibits p38β kinase to block downstream-located NF-κB-dependent signaling (Liang et al., 2018). As such, pharmacological inhibition of sEH to stabilize EETs may instigate PA vasoconstriction but somehow, mitigate pathological progression in the pulmonary circulation.

## Summary and Perspectives

We briefly summarized the pathophysiological significance of potentiating EET production and/or inhibiting EET hydrolysis, as a function of estrogen, in the regulation of systemic and pulmonary circulations. In the systemic circulation, increases in EETs afford better cardiovascular performance and lower incidence of ischemic diseases in women. In the pulmonary circulation, clinical development of PH appears to require “two hits” that can be triggered by either genetics (sex and gene polymorphisms), environmental factors (hypoxia and sex hormone dysmetabolism), or both (female with hypoxia). Thus, from a pros and cons point of view, targeting CYP and sEH may prove to be a double-edged sword with beneficial and adverse effects on systemic and pulmonary circulatory systems; this brings concerns surrounding the use of sEHIs as therapeutic regimens, or the consideration of sEHI-related clinical trials in female populations who bear hyper-responsiveness to acquired pathological insults (as second hits) to the respiratory system.

## Author Contributions

AH and DS contributed equally to the literature search, figures, and writing, and gave their final approval of the manuscript.

## Conflict of Interest Statement

The authors declare that the research was conducted in the absence of any commercial or financial relationships that could be construed as a potential conflict of interest.
